# Orbital Apex Syndrome Secondary to Retained Foreign Body: A Case Report

**DOI:** 10.7759/cureus.70210

**Published:** 2024-09-25

**Authors:** Anne M Umboh, Alberta Y Tansil, Rizki R Najoan

**Affiliations:** 1 Department of Ophthalmology, Faculty of Medicine, Sam Ratulangi University, Manado, IDN; 2 Department of Otolaryngology-Head and Neck Surgery, Faculty of Medicine, Sam Ratulangi University, Manado, IDN

**Keywords:** ophthalmoplegia, optic neuropathy, orbital apex syndrome, orbital foreign body, transnasal endoscopic surgery

## Abstract

Orbital foreign bodies, especially organic materials, can cause severe eye and orbital damage. Orbital apex syndrome (OAS), a complex condition affecting multiple cranial nerves, is often caused by trauma, among other factors. The patient was a three-year-old boy who fell onto a tree stump three days prior. He presented to the emergency department with left-sided eyelid edema, ptosis, traumatic mydriasis, numbness, and ophthalmoplegia and was diagnosed with OAS. Despite treatment with intravenous methylprednisolone, analgesia, and antibiotics, his condition did not improve after the transnasal endoscopic removal of the foreign body. This case highlights OAS caused by a wooden orbital foreign body requiring prompt, multidisciplinary surgical intervention. Early diagnosis and prompt intervention are crucial to preventing devastating outcomes like OAS and permanent visual impairment. Given the limited understanding of this condition, further research is essential to optimize management strategies and improve patient outcomes.

## Introduction

Orbital foreign bodies can inflict severe structural and functional damage on the eye and surrounding tissues. Organic foreign bodies can trigger inflammation and lead to critical complications [1]. Despite advancements in imaging technology, identifying and locating organic intraorbital foreign bodies remains challenging. Foreign bodies commonly invade the orbit by passing between the eyeball and its bony socket. However, they can also directly pierce the eye or enter from the adjacent facial sinuses [2].

Orbital apex syndrome (OAS) is a complex condition characterized by damage to the oculomotor nerve (CN3), trochlear nerve (CN4), an ophthalmic branch of the trigeminal nerve (CN5), and abducens nerve (CN6) and associated optic nerve (CN2) dysfunction [3,4]. Its etiology encompasses inflammation, infection, neoplasia, vascular abnormalities, trauma, and other factors [3-5]. The orbital apex is a confined space housing critical neurovascular structures, rendering it susceptible to damage, even from minor lesions. Traumatic OAS is uncommon and typically results from fractures, foreign body penetration, or bleeding within this region [6]. We report a case of OAS resulting from a wooden foreign body in the orbit that necessitated urgent, multidisciplinary surgical intervention.

## Case presentation

**Figure 1:**
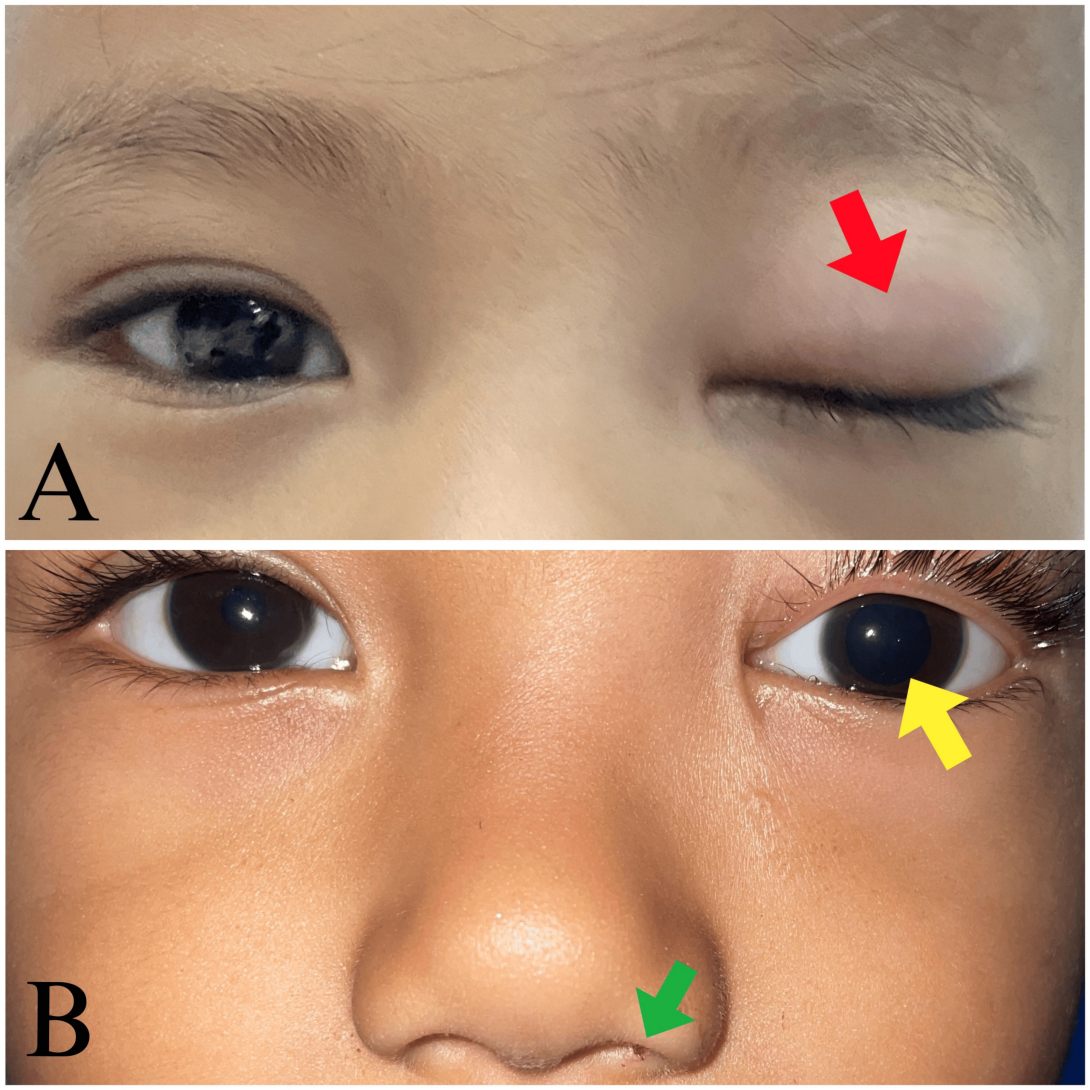
Clinical presentation of the patient (A) Left-sided eyelid edema (red arrow) and drooping upper eyelid. (B) A fixed and dilated pupil unresponsive to light with a positive relative afferent pupillary defect (yellow arrow) and left-sided epistaxis (green arrow)

A three-year-old boy presented to the emergency department at Kandou Hospital, Manado, three days after falling onto a tree stump. He had no history of syncope, vomiting, or seizures. On examination, he was alert and oriented. The parent reported removing a 1 cm wooden foreign body from his left nostril and nose bleeding. The patient has 6/6 visual acuity in the right eye and light perception in the left eye. Both eyes had normal eye pressure, measuring 11 mmHg in the right eye and 14 mmHg in the left. Ocular examination revealed left-sided eyelid edema, a fixed, dilated pupil unresponsive to light with a positive relative afferent pupillary defect (Figure 1), and numbness in his forehead, upper eyelid, and cornea. He had limited eye movement. The fundoscopic examination revealed no abnormalities.

**Figure 2:**
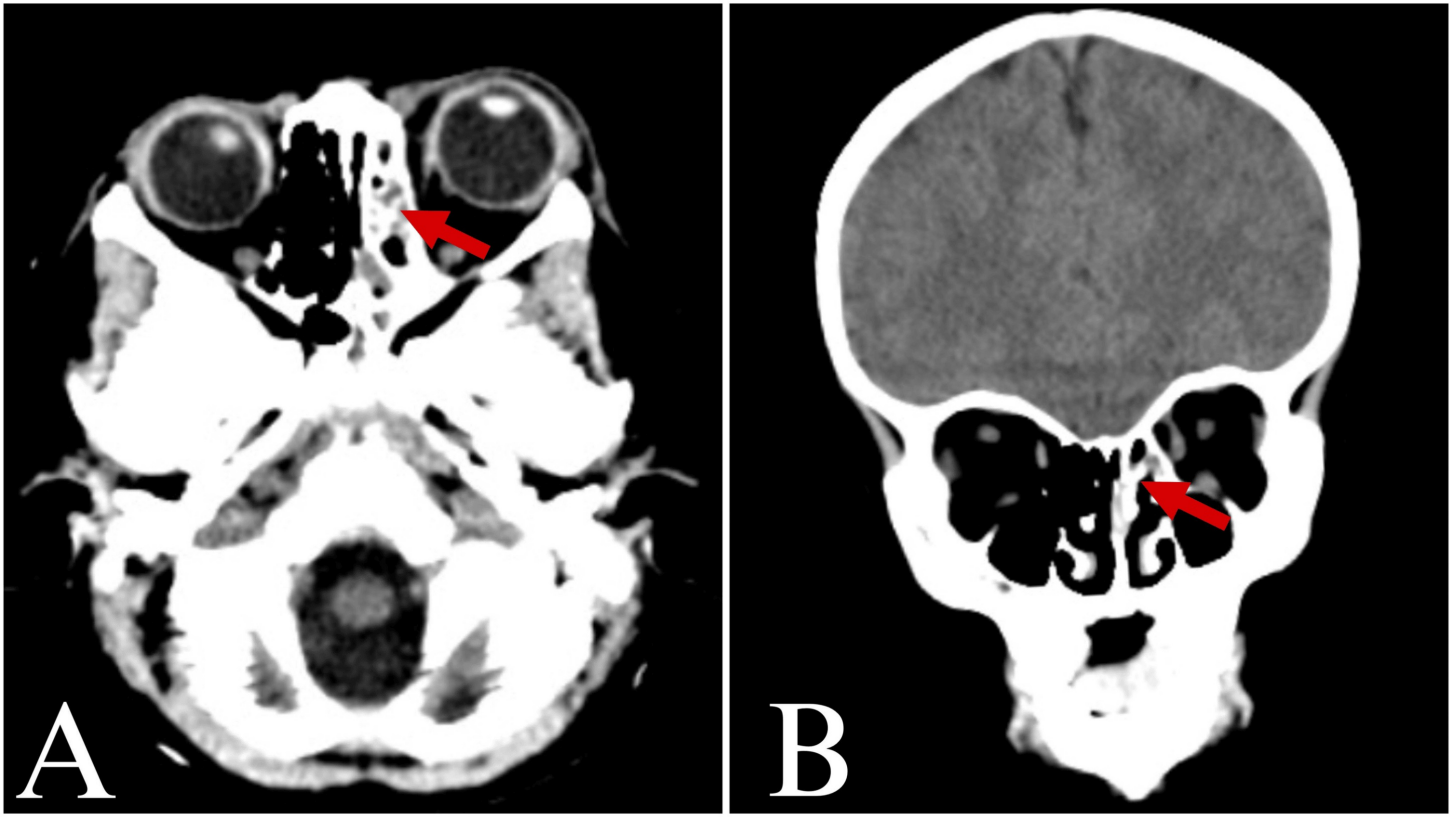
CT imaging demonstrated minimal orbital emphysema with associated left periorbital soft tissue edema (red arrow) (A) Coronal and (B) axial views of the head and orbits

A CT scan revealed minimal orbital emphysema and left periorbital soft tissue swelling. There were no signs of foreign bodies within the intraorbital or intracranial cavities (Figure 2).

**Figure 3:**
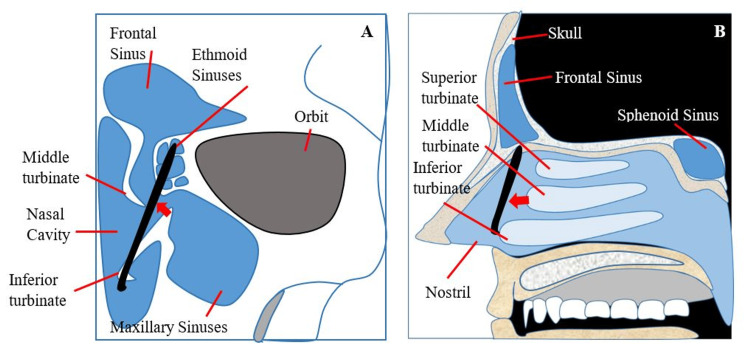
A simple diagram of a transnasal endoscopy procedure that shows the position of a foreign object (red arrow) (A) Axial view. (B) Sagittal view

The patient was diagnosed with OAS and treated with intravenous corticosteroids, analgesics, and antibiotics for five days. The ENT performed a transnasal endoscopy and found a foreign object located at the level of the middle turbinate, penetrating the middle turbinate and reaching the ethmoid sinus (Figure 3).

**Figure 4:**
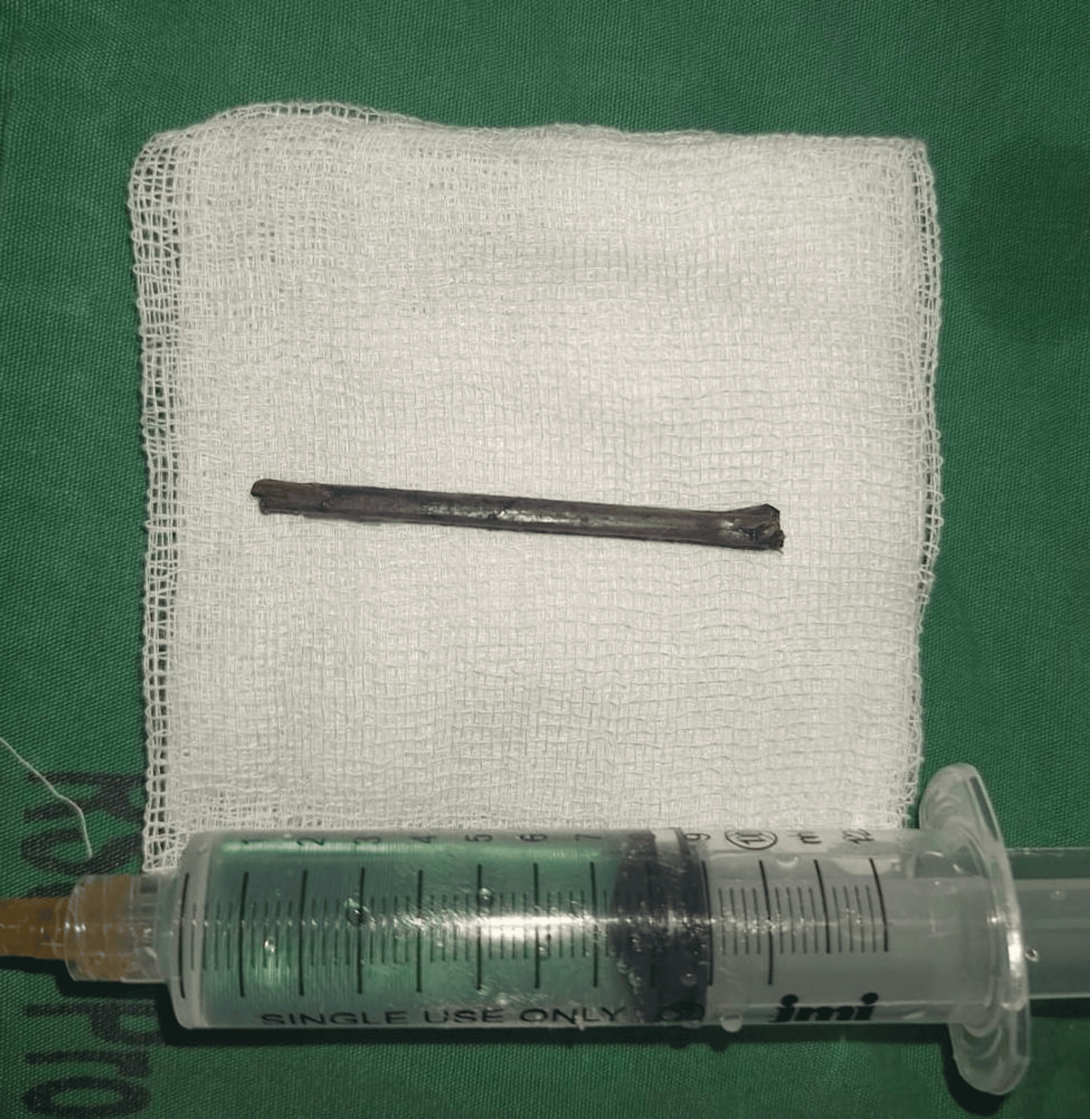
The wooden foreign body measured 43 mm in length

The foreign object was a 43 mm wooden piece (Figure 4). Despite the surgical intervention, the patient's ocular condition did not improve over the following seven days of inpatient care. Despite outpatient follow-up, the patient's delayed diagnosis resulted in irreversible eye damage and a poor clinical outcome.

## Discussion

OAS is a complex neurological condition characterized by optic neuropathy and ophthalmoplegia [1]. It occurs due to pathology within the orbital apex, a limited space housing crucial neurovascular structures. OAS is distinct from superior orbital fissure syndrome (affecting cranial nerves III, IV, VI, and V1 without optic neuropathy) and cavernous sinus syndrome (involving cranial nerves V2 and V3 and sympathetic fibers) [2].

OAS often presents with a combination of symptoms, including vision problems, restricted eye movements, pain, and proptosis. The causes of OAS are varied and can include inflammation, infection, tumors, vascular issues, and trauma [4]. Due to the confined anatomy of the orbital apex, even small lesions can have significant consequences. Traumatic OAS, although less common, can occur from bone fractures, penetration injuries, or bleeding. Foreign bodies lodged in this area, particularly within the orbital apex, pose a life-threatening risk to eye health and often require immediate medical attention to address potential vision loss. Organic foreign bodies, especially wood, are more prone to causing inflammation and infection compared to metallic objects [7,8]. Therefore, advanced imaging techniques like CT and MRI are crucial for accurately locating the object and evaluating the extent of any associated injuries [7].

Foreign objects can enter the orbit through various pathways, most commonly between the eyeball and orbital wall [9]. However, they can also penetrate through the eye or from the paranasal sinuses. The type of foreign body significantly affects the tissue's reaction, with irritating substances causing more severe inflammation than non-irritating materials. Fortunately, reported cases of traumatic OAS are rare [5,10,11]. Due to the limited number of cases, with traumatic superior orbital fissure syndrome occurring in less than 1% [5,12,13] and traumatic optic neuropathy ranging from 1% to 6% [13,14], treatment guidelines for traumatic OAS are still developing. Therefore, treatment often relies on knowledge gained from similar conditions like traumatic optic neuropathy [13].

Traumatic OAS may result from the compression of neurovascular structures as a result of direct impingement from an osseous fragment, foreign bodies, hemorrhage, or significant edema [13]. Gossman et al. previously identified two main mechanisms for traumatic optic neuropathy: direct and indirect. They classified direct traumatic optic neuropathy as injury to the optic nerve resulting from external pressure due to a fractured optic canal, as evidenced by radiological findings. Indirect injury was described as optic neuropathy without radiographic evidence of fracture or further proof of pathology along the course of the nerve [15].

The duration of irreversible ischemic damage to the optic nerve in OAS varies based on the extent of the injury. While studies suggest that damage can occur within an hour, the optimal time for intervention is still under debate. The critical nature of the situation necessitates immediate surgical assessment. Distinguishing between direct and indirect optic neuropathy can be difficult, especially in facial trauma cases with significant swelling, bleeding, or impaired blood flow. Both types can occur together, requiring a comprehensive treatment approach [13].

Clinical presentation is crucial in diagnosing OAS, with signs including anesthesia, external ophthalmoplegia, ptosis, fixed dilated pupil, lacrimal hyposecretion, diplopia, and decreased visual acuity. Damage or compression of the ophthalmic branch of the trigeminal nerve can result in numbness or loss of sensation in the cornea, upper eyelid, nose bridge, and forehead, potentially affecting the corneal reflex. Additional symptoms may include neuralgic pain in the first branch of the trigeminal nerve and pain behind the eye. External ophthalmoplegia, caused by impaired function of the oculomotor, trochlear, and abducens nerves, results in double vision. Ptosis occurs due to dysfunction of the levator palpebrae superioris and Müller's muscle. Decreased visual acuity is associated with optic neuropathy [10,16].

The optimal treatment for traumatic OAS remains controversial. Surgical intervention, such as endoscopic decompression, may be considered for patients unresponsive to high-dose corticosteroids despite their potential to improve vision [16]. It's important to note that while traumatic optic neuropathy is a related condition, the findings from the International Optic Nerve Trauma Study may not be directly applicable to OAS due to differences in the underlying pathology and anatomical structures involved [6]. The decision to use corticosteroids is complex. While they can reduce inflammation, microcirculatory spasms, edema, and nerve cell necrosis around the optic nerve, their risks and benefits must be carefully weighed. Surgical intervention is typically reserved for cases with clear evidence of nerve compression, such as from bony fragments or displaced orbital contents [16]. Despite receiving high-dose methylprednisolone, the patient's condition did not improve, and vision in the affected eye remained impaired. The delayed and progressive vision loss suggests ongoing optic nerve compression, likely from blood and edema. Earlier intervention might have improved the chances of recovery [16].

Removing foreign bodies from the orbital cavity presents a surgical challenge due to the confined anatomical space and limited surgical access [7,17]. Transnasal endoscopic surgery (TNES) has emerged as a valuable technique for addressing foreign bodies in the orbital apex. This minimally invasive approach offers improved visualization and reduces surgical invasiveness compared to traditional open surgical methods. Early studies have demonstrated the practicality of TNES without image-guided navigation. Nonetheless, the addition of navigation technology has markedly increased precision and minimized risks. While TNES offers numerous benefits, it's important to note that it may be associated with postoperative nasal discomfort, including crust formation, epistaxis, and discharge. TNES procedures can be time-consuming, but combined with image-guided navigation, they offer a promising approach for orbital apex foreign bodies [6,18]. In this case, a transnasal endoscopic evaluation identified a small wooden object embedded in the nasal septum. In this case, a transnasal endoscopic evaluation revealed and successfully removed a small wooden object lodged at the level of the middle turbinate without causing significant injury.

## Conclusions

OAS, a rare neurological condition characterized by optic neuropathy and ophthalmoplegia, necessitates prompt intervention when caused by foreign bodies lodged in the orbital apex. TNES provides a minimally invasive approach for removal, improving visualization and reducing surgical invasiveness. Corticosteroids and surgery are options, but their effectiveness depends on individual circumstances. Further research is essential to establish definitive treatment guidelines for traumatic OAS.
